# Examining the Utility of Social Media in COVID-19 Vaccination: Unsupervised Learning of 672,133 Twitter Posts

**DOI:** 10.2196/29789

**Published:** 2021-11-03

**Authors:** Tau Ming Liew, Cia Sin Lee

**Affiliations:** 1 Department of Psychiatry Singapore General Hospital Singapore Singapore; 2 Saw Swee Hock School of Public Health National University of Singapore Singapore Singapore; 3 SingHealth Polyclinics Singapore Singapore; 4 Family Medicine Academic Clinical Programme Duke-NUS Medical School Singapore Singapore

**Keywords:** social media, COVID-19, vaccine hesitancy, natural language processing, machine learning, infodemiology

## Abstract

**Background:**

Although COVID-19 vaccines have recently become available, efforts in global mass vaccination can be hampered by the widespread issue of vaccine hesitancy.

**Objective:**

The aim of this study was to use social media data to capture close-to-real-time public perspectives and sentiments regarding COVID-19 vaccines, with the intention to understand the key issues that have captured public attention, as well as the barriers and facilitators to successful COVID-19 vaccination.

**Methods:**

Twitter was searched for tweets related to “COVID-19” and “vaccine” over an 11-week period after November 18, 2020, following a press release regarding the first effective vaccine. An unsupervised machine learning approach (ie, structural topic modeling) was used to identify topics from tweets, with each topic further grouped into themes using manually conducted thematic analysis as well as guided by the theoretical framework of the COM-B (capability, opportunity, and motivation components of behavior) model. Sentiment analysis of the tweets was also performed using the rule-based machine learning model VADER (Valence Aware Dictionary and Sentiment Reasoner).

**Results:**

Tweets related to COVID-19 vaccines were posted by individuals around the world (N=672,133). Six overarching themes were identified: (1) emotional reactions related to COVID-19 vaccines (19.3%), (2) public concerns related to COVID-19 vaccines (19.6%), (3) discussions about news items related to COVID-19 vaccines (13.3%), (4) public health communications about COVID-19 vaccines (10.3%), (5) discussions about approaches to COVID-19 vaccination drives (17.1%), and (6) discussions about the distribution of COVID-19 vaccines (20.3%). Tweets with negative sentiments largely fell within the themes of emotional reactions and public concerns related to COVID-19 vaccines. Tweets related to facilitators of vaccination showed temporal variations over time, while tweets related to barriers remained largely constant throughout the study period.

**Conclusions:**

The findings from this study may facilitate the formulation of comprehensive strategies to improve COVID-19 vaccine uptake; they highlight the key processes that require attention in the planning of COVID-19 vaccination and provide feedback on evolving barriers and facilitators in ongoing vaccination drives to allow for further policy tweaks. The findings also illustrate three key roles of social media in COVID-19 vaccination, as follows: surveillance and monitoring, a communication platform, and evaluation of government responses.

## Introduction

COVID-19 first presented as an atypical pneumonia of unknown cause in Wuhan, China, in late December 2019. Within a short time frame, the virus spread to multiple countries despite international efforts to contain it, resulting in significant death, psychological impact, and economic disruption around the world [1]. This led the World Health Organization (WHO) to declare COVID-19 a Public Health Emergency of International Concern on January 30, 2020 [2], and a global pandemic on March 11, 2020 [3]. After more than one year of battling with the pandemic, vaccines for COVID-19 present as a promising and viable solution to end the pandemic, particularly through concerted efforts in global mass vaccination to achieve herd immunity from the COVID-19 virus [4]. On November 18, 2020, the hope of ending the COVID-19 pandemic became more conceivable when Pfizer-BioNTech announced in the press [5] the first available COVID-19 vaccine, which had 95% efficacy and a good safety profile [6].

Moving on, the next phase of the challenge was to ramp up public health efforts to increase vaccine uptake in the population, possibly through well-thought-out and coordinated vaccination drives. Yet, it is well known that vaccination drives are often hampered by the issue of “vaccine hesitancy,” whereby a large majority of population may have conflicting motivation or opposition to receive vaccines [7-10]. Historically, the issue of vaccine hesitancy has resulted in low coverage rates in adult vaccination programs [9]. For example, in the United States, vaccination coverage of the 2019-2020 influenza vaccine ranged from 48.4% among the adult population [11] to 80.6% among health care workers [12]. Similarly, in the context of COVID-19 vaccination, recent nationally representative surveys demonstrated that only 61.4% of the US population [13] and 64.0% of the UK population [14] were willing to receive COVID-19 vaccines. In the literature, many reasons have been cited for individuals’ conflicted motivation or opposition to receive vaccines [7-10]. Some of these reasons include concerns about vaccine safety, perceived low risk of contracting illness, perceived low severity of illness, fear of pain, perceived ineffectiveness of vaccines, and misinformation about vaccines [10]. Given the severity of the COVID-19 pandemic and the time pressure to accelerate COVID-19 vaccination, there is an urgent need to gain insight into the psychological proposition of how people think and feel—specific to the newly developed COVID-19 vaccines—so that effective recommendations or strategies may be proposed to improve vaccine uptake [7].

Traditionally, research on attitude, perception, and behavior has often relied on methodologies such as surveys, interviews, or focus group discussions. However, such traditional approaches can often be time-consuming in their data collection processes (ie, there can be a considerable time lag between the start of data collection and the eventual availability of data for further analysis), hence, findings from the traditional approaches may not sufficiently reflect real-time public sentiments on time-sensitive matters, such as those related to COVID-19 vaccination. An alternative would be using data from social media platforms such as Twitter, where researchers can collect near–real-time information that reflects prevailing perspectives and sentiments in the community [9,15,16]. Such an approach is in line with the growing field of infodemiology, which recognizes the utility of real-time information across the internet in informing public health and public policy [17,18]. The study of social media data is also particularly relevant in the context of COVID-19 vaccination, given recent findings that exposure to information, or misinformation, on social media can have a direct influence on individuals’ intentions to receive COVID-19 vaccines [9,19].

In this infodemiology study, we sought to examine public conversations about COVID-19 vaccines as posted on Twitter—specifically after the press release about the first effective vaccine by Pfizer-BioNTech on November 18, 2020—with the intention to shed light on the potential utility of social media in the context of the COVID-19 vaccination drive, as well as to identify useful strategies that may address COVID-19 vaccine hesitancy and improve vaccine uptake. Specifically, we intended to address the following two research questions:

1. What are the key issues that have captured public attention following the press release of the first effective COVID-19 vaccine?
2. How may social media data inform the barriers and facilitators that can influence individuals’ behaviors regarding receiving COVID-19 vaccines?

## Methods

### Data Source for the Infodemiology Study

Twitter was selected as the social media platform for data collection in this study, due to accessibility to researchers of its large quantity of global data [20]. Twitter is a popular social media platform worldwide [9], where members of the public may post their opinions and sentiments in short texts of up to 280 characters, also known as “tweets.” On a monthly basis, Twitter has an estimated 329.5 million active users from around the world [9]. It is recognized as the third most popular social media platform in the United Kingdom [21] and is used by one-quarter of people in United States [9]. All data used in this study were collected according to Twitter’s terms of use. The outline of the study was presented to the SingHealth Centralised Institutional Review Board of Singapore who concluded that the study did not meet the criteria for human subjects research requiring review by a research ethics committee.

A publicly available COVID-19 Twitter data set [20] was searched for original tweets (ie, not retweets or duplicate tweets) that were posted in English over an 11-week period from November 18, 2020 (ie, following the press release about the Pfizer-BioNTech vaccine) to February 3, 2021. The search terms were “COVID-19” (or similar terms, such as “coronavirus,” “corona virus,” “2019ncov,” “COVID,” “COVID19,” “COVID_19,” “COVID 19,” “CoronavirusPandemic,” “CoronaOutbreak,” and “WuhanVirus”) and “vaccine” (or similar terms, such as “vaccinat*,” “immuniz*,” “immunis*,” “innoculat*,” “anti-vaccin*,” “antivaccin*,” “anti_vaccin*,” “anti-vaxxer,” “antivaxxer,” and “anti_vaxxer”). Following the same approach used by Koh and Liew [22], only tweets that were posted by individual users, and not organizations or news outlets, were included in this study so that we could focus on individuals’ sentiments about COVID-19 vaccines and minimize the selection of objective reports about the vaccines, such as those in news articles, or tweets by nonhuman Twitter users (ie, bots). Individual Twitter users were identified by the use of actual human names on the Twitter account of each post; this process of identifying human names was conducted using the machine learning approach of natural language processing, based on the spacyr package [23] in R (version 4.0.2; The R Foundation).

### Unsupervised Learning of Free-Text Data From Twitter

As the tweets comprised large volumes of free-text data, the unsupervised machine learning approach of topic modeling was used to analyze these data [24]. Topic modeling is a machine learning technique that identifies key topics within free-text data, based on statistical probability and correlations among words. It is akin to thematic analysis in traditional qualitative methodology. But unlike thematic analysis, topic modeling does not require manual labor to classify free-text data and, hence, is well suited for analyses of large volumes of free-text data [24] such as in this study.

Before conducting topic modeling, the following steps were performed to preprocess the free-text data based on currently recommended best practices [22,24]. First, sentences from each tweet were tokenized (ie, reduced to single words, punctuation and extra spaces removed, and words converted into lowercase). Then, frequently occurring words that add little to the meaning of sentences were removed (eg, “the” and “a”). Next, the remaining words were converted to their root form (eg, “went” was transformed to “go,” and “friends” was transformed to “friend”). Lastly, words that occurred in less than 600 tweets (representing ~0.05% of tweets) were removed to reduce statistical noise and improve accuracy [24]. Preprocessing of free-text data was conducted using the spacyr package [23] in R.

In our topic modeling approach, the preprocessed words were presented to an unsupervised machine learning algorithm to identify clusters of words that tended to co-occur together. Words with a high probability of co-occurring in tweets were then considered to belong to the same “topic.” Three covariates were included in topic modeling (ie, continent of tweets, date of tweets, and number of followers of the Twitter users) to improve the accuracy of the model in identifying topics. The optimal number of topics was identified using the algorithm proposed by Lee and Mimno [25]. Topic modeling was conducted using the stm package [26] in R.

### Thematic Analyses to Further Refine the Output From Unsupervised Learning

Output from topic modeling was examined by the two authors (TML and CSL) to ensure coherence of the identified topics. A descriptive label for each topic was manually crafted by the two authors based on sample tweets of each topic. Thereafter, the topics were further grouped into themes by the two authors using the inductive and iterative processes of thematic analysis as introduced by Braun and Clarke [27].

In addition to the inductive thematic analysis, we also adopted the COM-B (capability, opportunity, and motivation components of behavior) model [28] to guide our analysis for the second research question: “How may social media data inform the barriers and facilitators that can influence individuals’ behaviors in receiving COVID-19 vaccines?” The COM-B model was previously developed to provide a framework to understand and change human behaviors in the context of public health [28]. It has been widely used in the literature to understand human behavior in areas such as medication adherence [29], smoking cessation [30], diabetes [31], and obesity [32], and has recently been adopted by Public Health England as a key framework to guide its policy making [33]. Specific to the area of vaccine hesitancy, the COM-B model has been successfully applied in the literature to understand the barriers and facilitators related to childhood vaccination [34] and human papillomavirus vaccination [35]. In essence, the COM-B model proposes that an individual’s behavior is the result of an interaction between three components: capability, opportunity, and motivation. Capability refers to an individual’s psychological and physical capacity to make the behavior possible, such as having the necessary knowledge and skills to perform the target behavior. Opportunity refers to attributes that lie outside the individual physically or socially that make the behavior possible, such as environmental factors or social and cultural norms. Motivation refers to the automatic or reflective mental processes that energize and direct behavior, which can include the conscious thought process in deciding on a behavior or a less conscious thought process driven by desires or habits. By conceptualizing human behaviors using the three components (ie, capability, opportunity, and motivation), the COM-B model allows policy makers to design evidence-based interventions that specifically target each of the components [28]. Some examples are as follows:

- Issues related to capability can often be modified through education and training [28].
- Issues related to opportunity may possibly be modified through environmental restructuring (ie, shaping the physical or social environment to constrain or promote behavior), enablement (ie, providing the right support or tool to facilitate a desirable behavior), and restriction (ie, using rules to reduce the opportunity to engage in an undesirable behavior) [28].
- Issues related to motivation may potentially be modified, for example, through communication, modeling (ie, providing an example for people to aspire to or imitate), and incentivization (ie, creating an expectation of reward) [28].

### Sentiment Analysis of Free-Text Data

To further enrich the main findings, we conducted an exploratory analysis to identify the underlying emotions of each tweet using the VADER (Valence Aware Dictionary and Sentiment Reasoner) package [36] in R. VADER is an established sentiment analysis tool that has been widely employed in recent studies of free-text data on Twitter [37-40] and online news [41]. For each tweet, VADER used a rule-based machine learning model to identify three key emotions (ie, positive, negative, and neutral), which were then combined into a composite sentiment score ranging from –1 (most negative sentiment) to +1 (most positive sentiment). The sentiment score for each topic was then computed by averaging the sentiment scores of all the tweets within the topic.

Although many sentiment analysis tools are currently available [36,42-45], VADER offers some advantages over existing tools. First, it is specifically attuned to sentiments expressed in social media [36] and has been extensively validated for Twitter content [36,42,44].

Second, it is based on a human-curated lexicon of 7500 emotion-related words as well as five human-interpretable rules that identify sentiment intensity (ie, exclamation point, capitalization, intensity adverbs, the contrastive conjunction “but,” and negation flips preceding each emotion-related word). As such, unlike other sophisticated machine learning models, VADER’s classification rules are interpretable by humans and not hidden within a machine-access-only black box [36,43].

Third, as VADER uses a simple rule-based machine learning model, it is computationally efficient and only takes a fraction of computational time to analyze free-text data compared to other sophisticated machine learning techniques. Using an example from a previous study [36], a set of free-text data that took less than a second to analyze with VADER could take hours when using more complex models, such as the support vector machine model.

Fourth, as VADER is based on a human-validated lexicon, it does not require any form of training data. This is in contrast to other sophisticated machine learning models that often require extensive sets of training data before they can produce accurate results in sentiment analysis [36,43].

Fifth, despite its simplicity, VADER has been shown in several comparative studies [36] to outperform many other highly regarded sentiment analysis tools. In one of the initial validation studies [36], VADER was found to have an overall accuracy of 96% in identifying correct sentiments in tweets. It was considerably better than seven other well-established sentiment analysis lexicons (ie, Linguistic Inquiry Word Count, General Inquirer, Affective Norms for English Words, SentiWordNet, SenticNet, Word-Sense Disambiguation using WordNet, and Hu & Liu Opinion Lexicon; overall accuracy of 56%-77%) and four other machine learning algorithms (ie, naïve Bayes, maximum entropy, support vector machine–classification, and support vector machine–regression; overall accuracy of 65%-84%). Notably, in the same validation study [36], VADER was also shown to outperform individual human raters which had an overall accuracy of 84%. Similar findings were replicated in more recent comparative studies [42,43], with VADER outperforming four sentiment analysis tools (ie, SentiWordNet, SentiStrength, Hu & Liu Opinion Lexicon, and AFINN-111) in one study [42] and two other tools (ie, TextBlob and Natural Language ToolKit) in another study [43].

Lastly, VADER is available as an open-source package that is easily accessible from widely used data science platforms, such as R and Python.

## Results

### Identification of Topics From Tweets

**Figure 1:**
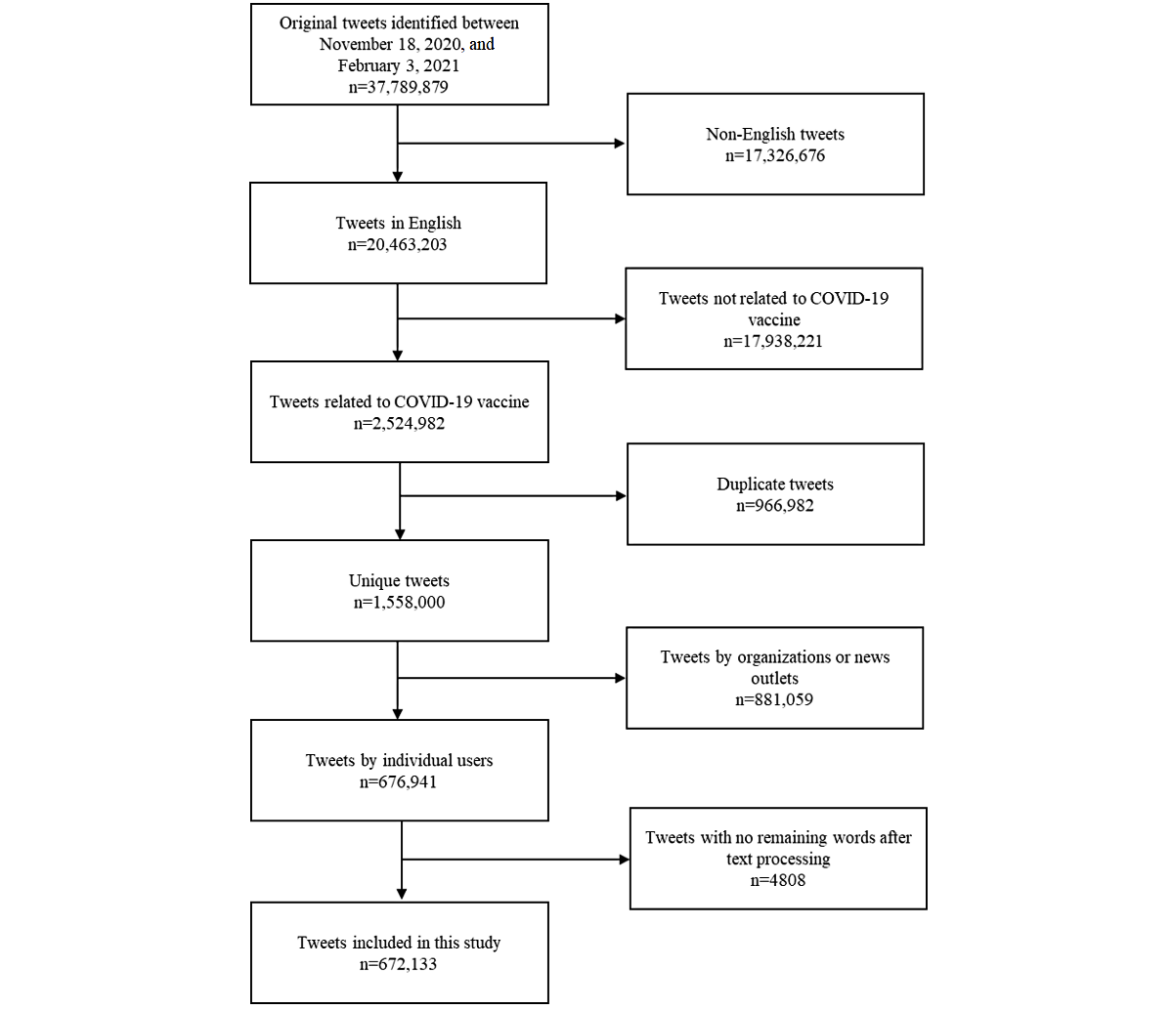
Flow diagram showing the selection of Twitter posts for this study.

**Figure 2:**
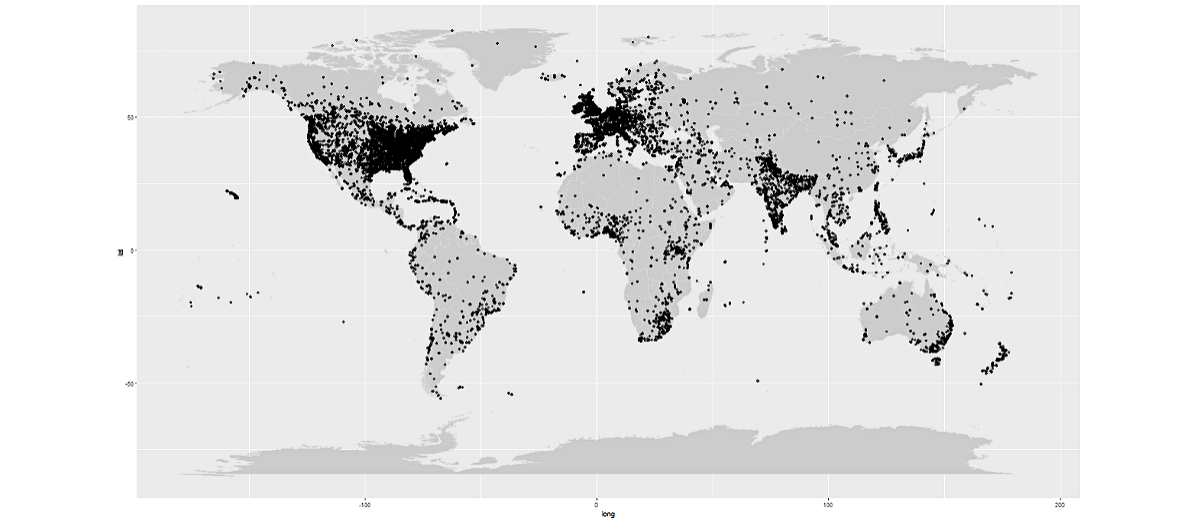
Geographical distribution of the Twitter posts. lat: latitude; long: longitude.

A total of 2,524,982 tweets were initially identified over the 11-week study from November 18, 2020, to February 3, 2021. After removing duplicate tweets and tweets from news media or organizations, a total of 672,133 tweets were eventually included. A flow diagram showing tweet selection is presented in Figure 1. The geographical distribution of tweets is shown in Figure 2, with 38.5% originating from North America, 14.3% from Europe, 5.7% from Asia, 2.1% from Africa, 1.4% from Australia, 1.1% from South America, and 37.0% from unknown locations.

Using the unsupervised machine learning algorithm of topic modeling, 60 topics could initially be identified from the tweets. Coherence of the 60 topics was manually examined by the two authors; during this process, one topic that had the lowest prevalence (~0.4%) was found to bear much similarity with another topic and, hence, the two topics were combined. As such, a total of 59 topics were eventually included. Following thematic analysis by the two authors, the 59 topics could be further grouped into six themes. Word clouds for the six themes are shown in Multimedia Appendix 1, while details related to each topic are presented in Table 1 as well as further described in the following paragraphs.

**Table 1:** Six themes related to COVID-19 vaccination, along with the respective topics and sample tweets (N=672,133).

Theme and topics (keywords)			Sample tweet		Prevalence, %		Sentiment score^a^
**Theme 1: emotional reactions related to COVID-19 vaccines**							
	Topic 37: feeling hopeful after receiving COVID-19 vaccines (be, shot, get, feel, go, ready, see, happy, hope, and friend)	“Cried happy tears seeing family and friends abroad getting their COVID19 vaccines. Better days ahead”		3.2		+0.262	
	Topic 30: feeling excited after receiving COVID-19 vaccines (get, receive, give, do, administer, complete, delay, deliver, waste, and order)	“Perks of completing the COVID19 vaccine series... we got a sticker!!.”		2.6		+0.097	
	Topic 18: feeling angry toward politicians for claiming COVID-19 as a hoax (think, people, anti, right, believe, know, hoax, should, thing, and politician)	“Wow, wow majority of Republican house and senate are vaccinated. They are the one who denied that coronavirus existed and is a hoax. My, my they are the first one in the queue. They should not be in the queue at all if they believe coronavirus is a hoax.”		2.6		–0.109	
	Topic 17: feeling angry toward President Trump for claiming COVID-19 as a hoax (want, go, would, Trump, will, name, Americans, credit, let, and people)	“The scientists developed the vaccines not the Trump Administration. The Trump Administration is responsible for 300,000+ deaths of Americans due to their incompetency and lying about the Coronavirus for months. Trump called the virus a Democratic HOAX!”		2.3		–0.110	
	Topic 42: feeling thankful toward scientists who were involved in the development of COVID-19 vaccines (news, thank, end, day, science, Moderna, team, work, Pfizer, and job)	“Thank you thank you thank you for my covid-19 vaccine today. I feel extremely lucky and grateful. Thank you to the scientists and everyone behind rolling out the vaccine. In a world of darkness there is light at the end of the tunnel”		2.2		+0.482	
	Topic 59: plea to stay vigilant even with the rollout of COVID-19 vaccination (mask, keep, continue, stay, social, wear, hand, safe, distance, and wearamask)	“Please remember control of COVID is in our hands- hands that put on our masks, the hands we sanitize and hands to maintain our spacing. Vaccines are coming but we cannot let our collective guard down!”		2.2		+0.175	
	Topic 40: feeling proud to have contributed to the development of COVID-19 vaccines (part, share, late, thank, volunteer, experience, involve, learn, help, and successful)	“We’re proud to have played a part in the successful development of COVID19 vaccine.”		1.5		+0.409	
	Topic 33: feeling frustrated about the inequitable access of COVID-19 vaccines (should, person, last, member, chief, resident, mandatory, people, outbreak, and medical)	“‛r there no workhouses?? r there no prisons??’ Prisons are Covid-19 hotbeds. When should inmates get the vaccine?”		1.4		–0.028	
	Topic 55: feeling angry over the antivaccine movement and conspiracy claims (claim, conspiracy, misinformation, Twitter, dangerous, theory, medium, true, truth, and Bill_Gates)	“Sad that there even have to be articles like this to rebut the right-wing and anti-vaxer nonsense: No, COVID-19 vaccines don’t contain Satan’s microchips (and other scary conspiracy theories aren’t true either)”		1.4		–0.188	
**Theme 2: public concerns related to COVID-19 vaccines**							
	Topic 19: concerns about death related to COVID-19 vaccines (people, die, many, more, kill, number, infect, most, death, and will)	“People are known to die from so many illnesses. Millions die each day, so many die suddenly. Imagine people who were supposed to die suddenly on a particular day having recieved Covid-19 vaccine on the preceding day, many of them will try to blame Covid-19 vaccine”		2.3		–0.241	
	Topic 11: concerns about the impact of COVID-19 vaccines on fertility (mRNA [messenger RNA], work, safety, efficacy, base, explain, research, datum, understand, and thread)	“It is UNKNOWN whether COVID-19 mRNA Vaccine BNT162b2 has an impact on fertility. Animal studies into potential toxicity to reproduction and development have not been completed”		2.1		+0.236	
	Topic 26: concerns about misinformation related to COVID-19 vaccines (good, great, news, big, bad, fake, break, welcome, sell, and wake)	“Fed up of: lies lockdown FAKE NEWS covid vaccine coronavirus”		1.8		+0.206	
	Topic 12: concerns about insufficient supply of COVID-19 vaccines (supply, have, run, hold, problem, try, demand, due, lack, and could)	“New York City could run out of COVID19 vaccines as early as next week. We need the federal government to act NOW.”		1.6		–0.057	
	Topic 31: concerns about delays in vaccine rollout amid rising deaths related to COVID-19 (death, case, story, record, rollout, top, more, rise, and us)	“The US set a new record for coronavirus deaths reported in 24 hours with more than 3,700 on Wednesday amid troubles with the vaccine rollout. The harrowing tally from Johns Hopkins University marked the fourth time daily deaths have exceeded 3,000 throughout the pandemic.”		1.5		–0.027	
	Topic 54: concerns about vaccine scams that mine personal and financial details (important, information, warn, send, detail, info, scam, write, fall, and more)	“scam alert service has notified of a scam COVID-19 vaccine text linking to a bogus NHS website with a registration form for the vaccine in an attempt to mine personal and financial details.”		1.5		+0.141	
	Topic 43: concerns about the safety of COVID-19 vaccines among pregnant women and children (should, woman, become, child, scientist, risk, man, decision, pregnant, and benefit)	“They have not experimented the COVID19 vaccine on pregnant women and young children.”		1.4		+0.078	
	Topic 5: concerns about the reported statistics on adverse events of COVID-19 vaccine (report, reaction, cause, serious, severe, history, allergic, event, adverse, case)	“USA death rates: COVID-19 cases VS reported adverse events (CDC link) Specific rate: VACCINE MODERNA VACCINE FATALITY REPORT EVENT RATE: 5.82% COVID-19 CASE FATALITY RATE: 1.7%”		1.3		–0.243	
	Topic 6: concerns about fitness to receive COVID-19 vaccines among individuals with medical conditions or allergies (know, read, jab, issue, need, have, people, rush, should, and article)	“Info so far, COVID19 vaccine is NOT a danger for people with allergies to foods or medications”		1.3		–0.004	
	Topic 16: concerns about the short development phases of COVID-19 vaccines (flu, virus, cold, cure, common, different, cancer, sense, immunity, make)	“40 years research to find a cure for HIV AIDS.. still no vaccine. More than 100 years research to find a cure for cancer... still no vaccine. Ongoing research to find a cure for common cold and flu. Less than a year of Covid19 and you have a vaccine? No thanks! logicalthinking”		1.3		–0.060	
	Topic 53: concerns that the vaccines would not be effective against a new strain of the COVID-19 virus (new, strain, post, case, virus, variant, will, mutate, lockdown, and year)	“New Strains Of COVID Could Render Vaccines Completely Useless, And 2 Dangerous New Strains Are Already Spreading via COVID19”		1.3		–0.029	
	Topic 50: concerns about the short-term and long-term side effects of COVID-19 vaccines (effect, side, long, term, possible, potential, know, will, may, and short)	“COVID19 vaccines: What are the potential side effects? Doctors say most people will experience side effects such as fatigue, a sore arm for a day or two. Long-term effects are still unknow”		1.2		–0.023	
	Topic 41: concerns about COVID-19 vaccines causing false positive results on HIV tests (test, positive, antibody, HIV, virus, testing, rapid, quarantine, symptom, and negative)	“This is completely false and misrepresented. The vaccines didn’t CAUSE people to get HIV, it caused them to have false-positive results. Totally different. Why an Australian COVID-19 vaccine caused false-positive HIV tests”		1.2		+0.147	
**Theme 3: discussions about news related to COVID-19 vaccines**							
	Topic 32: news about the launch of the COVID-19 vaccination drive in India (country, India, world, start, drive, will, nation, program, large, and poor)	“India is one of the world’s largest vaccine manufacturers. India has started one of the world’s largest mass vaccinations”		2.6		+0.133	
	Topic 1: news about Oxford-AstraZeneca COVID-19 vaccine approval (UK, EU, roll, approve, AstraZeneca, Pfizer_, Britain, Oxford, country, and Europe)	“Cheapest Coronavirus Vaccine: UK Approves Oxford-AstraZeneca Jab, Rollout to Begin from Jan 4. OxfordAstraZeneca By far the cheapest vaccine.”		1.9		+0.139	
	Topic 52: news about the effectiveness of the vaccines against new strains of the COVID-19 virus (new, may, variant, mutation, current, virus, spread, appear, find, and suggest)	“CNN: A new study provides early evidence that a Covid-19 vaccine might be effective against two new coronavirus variants first identified in South Africa and the UK, despite a concerning mutation.”		1.9		+0.074	
	Topic 10: news about the approval of the Pfizer-BioNTech COVID-19 vaccine in the United States (use, Pfizer, approve, FDA, approval, authorization, Moderna, authorize, BioNTech, and seek)	“The FDA officially approves the Pfizer-Biontech coronavirus vaccine, the first approved in the U.S.”		1.7		+0.135	
	Topic 13: news about progress in the development of various COVID-19 vaccines (trial, phase, show, result, datum, clinical, human, study, candidate, and efficacy)	“still in phase 3 clinical trials. There are now about 12 COVID-19 vaccines in phase-3, only Pfizer, Moderna and Gamaleya have released interim efficacy results. Even AstraZeneca which was one of the first to enter phase-3 have not released efficacy results.”		1.7		+0.150	
	Topic 25: news about the approval of the Moderna COVID-19 vaccine in the United States (recommend, clear, US, FDA, vote, meet, panel, business, Moderna, and CEO)	“US clears second vaccine by Moderna for COVID-19: US clears Moderna vaccine for COVID-19”		1.2		+0.155	
	Topic 23: news about the approval of the Pfizer-BioNTech COVID-19 vaccine outside of the United States (emergency, BioNTech, Pfizer, approve, drug, UK, Moderna, could, worry, and the_United_States)	“Canada also approves COVID19Vaccine. Health Canada officially approves the Pfizer-BioNTech COVID-19 vaccine”		1.1		+0.126	
	Topic 56: news about the impact of COVID-19 around the world (doctor, NHS, policy, health, today, insurance, retweet, biotech, patients, and todaysmedicalupdate)	“As the virus resurges, mental health woes batter France”		0.8		+0.198	
	Topic 35: general news related to COVID-19 (medicine, pharmaceutical, medtech, politic, industry, breakingnews, FoxNews, health, giant, and science)	“California Reports First Case Of New COVID Variant”		0.5		+0.065	
**Theme 4: public health communications about COVID-19 vaccines**							
	Topic 8: involving an expert panel in public education on COVID-19 vaccines (expert, answer, join, discuss, Dr, concern, regard, key, check, and address)	“On this special episode, we talk with Dr. Steve Rockoff from Henry Ford West Bloomfield and Dr. Russell Faust from Oakland County Health as they answer FAQs about the COVID19 vaccine”		1.6		+0.258	
	Topic 9: using radio as a medium to clarify questions related to COVID-19 vaccines (question, ask, lot, wonder, talk, take, will, listen, hear, and come)	“Have a question about Covid or the vaccine? Send them my way and I'll ask as many as I can to our experts on-air!”		1.6		+0.139	
	Topic 22: using video to explain how COVID-19 vaccines work (patient, video, staff, watch, check, fact, live, link, email, message)	“Loved youtube videos on the Covid-19 vaccine! For those who are unsure and want to learn about how the covid-19 vaccine works please watch the video below!”		1.5		+0.213	
	Topic 34: summarizing the evidence on the efficacy of the Johnson & Johnson vaccine (effective, safe, prevent, disease, infection, show, single, Johnson, severe, and say)	“BREAKING: Johnson & Johnson says its single-shot covid-19 vaccine is: - 66% effective against moderate disease - 85% effective against severe disease”		1.5		+0.276	
	Topic 51: summarizing the evidence for the efficacy of the Sinovac vaccine (China, develop, produce, buy, Russia, Chinese, AstraZeneca, deal, Brazil, and purchase)	“An experimental COVID-19 vaccine developed by Chinese biopharmaceutical company Sinovac is 91.25% effective, a Turkish health official said on Thursday.”		1.5		+0.094	
	Topic 7: providing simple explanations on how COVID-19 vaccines can help in achieving herd immunity (life, will, save, change, immunity, stop, end, cost, herd, and affect)	“The vast majority of vaccinated people will be protected from contracting Covid-19 at all. In turn, the virus will have far fewer opportunities to spread through the population (think a forest that’s 90% firebreaks) and so transmission will be massively reduced.”		1.3		+0.115	
	Topic 20: providing simple explanations about how mRNA vaccines work (system, immune, virus, body, make, future, will, DNA, cell, mind)	“The vaccine will not contain any virus. It will only contain the instructions (mRNA) on how to build those spike proteins. Your body then learns how to recognize those spikes so that your immune system can kill coronavirus.”		1.3		+0.104	
**Theme 5: discussions about the approach to COVID-19 vaccination drives**							
	Topic 14: involving public figures to gain public trust regarding COVID-19 vaccines (take, say, public, available, trust, refuse, people, Americans, and would)	“I trust it and will take the vaccine when available! Obama, Bush and Clinton say they will take the COVID-19 vaccine publicly to gain public trust”		3.2		+0.154	
	Topic 48: using an appointment system to register for COVID-19 vaccination (appointment, call, sign, make, old, schedule, clinic, eligible, senior, and register)	“Florida has launched a statewide pre-registration system for individuals who are eligible for the COVID-19 vaccine. You can pre-register for appointments and be notified when appointments are available in your area by visiting”		2.5		+0.100	
	Topic 24: involving employers to facilitate COVID-19 vaccination (can, wait, require, travel, make, employee, return, employer, mandate, and proof)	“Companies are considering compulsory COVID19 vaccination as a condition of employment. ‛Under the law, an employer can force an employee to get vaccinated, and if they don’t, fire them,’ said Rogge Dunn, a Dallas labor and employment attorney.”		2.1		+0.076	
	Topic 45: clarifying the subpopulations to prioritize for COVID-19 vaccines (care, health, school, home, teacher, worker, access, risk, include, and staff)	“When the vaccine is rolled out and the elderly especially in long term care and all health care workers are vaccinated, teachers should be soon after. If face to face brick and mortar schools are considered essential, teachers, education workers and school staff must be soon after.”		1.8		+0.153	
	Topic 28: providing COVID-19 vaccines to frontline workers (worker, line, healthcare, frontline, front, next, essential, cut, stand, and place)	“COVID19 vaccine now for all frontline healthcare workers - patient care workers, no matter their title, deserve it before administrators.”		1.7		+0.103	
	Topic 4: prioritizing COVID-19 vaccines for long-term care homes (health, care, home, facility, will, resident, roll, worker, official, and minister)	“The Province will begin COVID-19 vaccinations of health-care and long-term care workers at hospitals in Toronto and Ottawa starting Dec. 15”		1.6		+0.209	
	Topic 46: defining high-risk health conditions to prioritize for COVID-19 vaccines (group, free, priority, list, pay, should, people, citizen, general, and give)	“Should smoking be considered a high risk condition and smokers have priority for CovidVaccine?”		1.5		+0.109	
	Topic 36: addressing mistrust among minority communities with regard to COVID-19 vaccination (community, challenge, leader, fear, black, role, hesitancy, equity, approach, and market)	“As MDs, it is crucial that we discuss this sensitive issue among our vulnerable minority communities, esp because it has/is still annihilating Black/Latinx communities. We must first acknowledge the history of medical mistrust.”		1.4		+0.210	
	Topic 58: showing COVID-19 vaccination of public figures on live television (president, live, receive, nurse, Dr, Biden, watch, elect, Joe_Biden, and former)	“US president-elect Joe Biden receives Pfizer’s Covid-19 vaccine shot on live television”		1.4		+0.144	
**Theme 6: discussions about the distribution of COVID-19 vaccines**							
	Topic 27: updates on the number of people who have received COVID-19 vaccines (dose, say, receive, Pfizer, administer, people, arrive, accord, distribute, and first)	“To date, 7,761 doses of the COVID-19 vaccine have been administered in Connecticut. Last week, we received 31,200 doses of Pfizer’s vaccine and anticipate receiving another 24,375 of that vaccine this week.”		2.7		+0.096	
	Topic 15: call for equitable access to COVID-19 vaccines across different countries (need, help, protect, ensure, work, must, bring, can, let, and access)	“No one is safe until everyone is safe COVAX: Ensuring global equitable access to COVID-19 vaccines”		2.1		+0.297	
	Topic 38: discussions about the distribution plan of COVID-19 vaccines in the United States (Trump, company, administration, speed, development, delivery, promise, distribution, effort, and US)	“The federal ‛Operation Warp Speed’ calls on multiple organizations and companies in the logistics of distributing of millions of COVID-19 vaccines.”		2.0		+0.062	
	Topic 57: call for a coordinated distribution plan of COVID-19 vaccines (plan, distribution, Biden, pandemic, strategy, rollout, national, release, relief, and economic)	“Decentralization of the distribution of vaccine has left a patchwork of madness. Vaccine distribution must be a National plan with the distribution centralized with the military. This is war - fight Covid like a war.”		2.0		+0.096	
	Topic 47: comparing the statistics related to COVID-19 and COVID-19 vaccination in the United States (state, number, rate, governor, track, California, slow, rollout, total, and case)	“Maryland coronavirus cases 1/18 328,214 0.5% higher than day before (+1769) Deaths 6423 0.45% higher than day before (+29) Hospitalizations day over day ICU +13 (421) Acute +14 (1429) Vaccines day over day +9569 (233,309)”		1.8		+0.023	
	Topic 49: updates on the shipment of COVID-19 vaccines (dose, hospital, expect, begin, shipment, Pfizer, will, arrive, breaking, and batch)	“UPS and FedEx trucks carrying the first U.S. shipment of coronavirus vaccine have left Pfizer’s facility near Kalamazoo, Michigan.”		1.7		+0.056	
	Topic 21: call for equitable access to COVID-19 vaccines across different income groups (would, could, Trump, like, god, time, election, American, money, and America)	“The Elite & Wealthy truly SUCK! The wealthy scramble for COVID-19 vaccines: ‛If I donate $25,000 ... would that help me?’ Vaccine Sad Wealthy covid CovidVaccine COVID19”		1.6		+0.153	
	Topic 44: updates on the rollout of the COVID-19 vaccination plan in Canada (Canada, government, will, force, minister, cdnpoli [Canadian politics], say, Ontario, head, and restriction)	“The Canadian military says it will be ready to distribute COVID-19 vaccines At noon, federal officials are providing an update on Canada’s vaccination plan”		1.6		+0.039	
	Topic 39: call for equitable access to COVID-19 vaccines across different ethnic groups (population, world, lead, Israel, provide, race, corona, power)	“Human Rights Watch has called on ‛Israel’ to provide COVID-19 vaccines to more than 4.5 million Palestinians in the occupied West Bank and Gaza Strip. HRW urges ‛Israel’ to provide COVID vaccines to Palestinians”		1.5		+0.035	
	Topic 29: updates on the rollout of COVID-19 vaccination sites (site, open, mass, testing, close, centre, major, city, injection, and turn)	“Mass vaccination sites soon will be added to Chicago’s mass testing sites COVID19”		1.4		+0.075	
	Topic 3: guidelines on the ethical considerations in the allocation of COVID-19 vaccines (follow, CDC [Centers for Disease Control and Prevention], meeting, director, guidance, guideline, recommendation, publichealth, allocation, and update)	“In addition to scientific data and implementation feasibility, four ethical principles will assist ACIP in formulating recommendations for the initial allocation of COVID-19 vaccine: 1) maximizing benefits and minimizing harms; 2) promoting justice;”		1.0		+0.107	
	Topic 2: comparing the statistics related to COVID-19 and COVID-19 vaccination outside of the United States (high, low, economy, reduce, contract, risk, price, mortality, and pandemic)	“Italy seems close to rise again in its COVID19 epidemic activity (R-eff=1.07), at high levels of activity, plateauing at very high levels of mortality, for 7 more days. 128,880 vaccinated as of Jan 04.”		0.9		+0.042	

^a^The sentiment scores range from –1 (most negative sentiment) to +1 (most positive sentiment).

### Descriptions of Six Identified Themes

Theme 1 describes “emotional reactions related to COVID-19 vaccines,” and involved 19.3% of the tweets. Tweets in this theme had a mixture of negative emotions, including those toward politicians, over inequitable access of vaccine, and over the antivaccine movement, as well as positive emotions, including those regarding receiving the vaccines and availability of the vaccines. Theme 2 describes “public concerns related to COVID-19 vaccines,” and involved 19.6% of the tweets. Common concerns included vaccine-related death, impact of the vaccines on fertility, and widespread misinformation related to the vaccines. Meanwhile, concerns with a negative tone included those related to insufficient supply of vaccine, delays in vaccine rollout, reported statistics on adverse events related to the vaccines, fitness to receive the vaccines among individuals with medical conditions or allergies, short development phases regarding the vaccines, effectiveness of the vaccines against new strains of the COVID-19 virus, and short-term and long-term side effects of the vaccines.

Theme 3 describes “discussions about news related to COVID-19 vaccines,” and involved 13.3% of the tweets. Commonly discussed news items included those related to vaccine development and approval as well as effectiveness of the vaccines against new strains of the COVID-19 virus. Theme 4 describes “public health communications about COVID-19 vaccines,” and involved 10.3% of the tweets. Tweets in this theme included efforts to provide simple explanations on vaccine-related issues, such as those related to the vaccines’ efficacies and mechanisms of action, as well as the use of various media in providing public education (eg, involving expert panels, radio, and video).

Theme 5 describes “discussions about the approach to COVID-19 vaccination drives,” and involved 17.1% of the tweets. Within this theme, several approaches to vaccination drives have commonly been highlighted, including engaging relevant stakeholders in vaccination drives (eg, public figures, employers, and minority communities) and defining priority groups for vaccination (eg, frontline workers, long-term care homes, and individuals with high-risk health conditions). Theme 6 describes “discussions about the distribution of COVID-19 vaccines,” and involved 20.3% of the tweets. Commonly described topics in this theme included discussions about equitable access to vaccines, such as ethical principles of vaccine allocation and equitable access across various subpopulations, as well as rollout efforts of COVID-19 vaccination (eg, shipment, distribution plan, vaccination sites, and vaccination statistics).

### Barriers and Facilitators to COVID-19 Vaccination

We further examined the barriers and facilitators related to COVID-19 vaccination by matching our initial 59 topics to the three components in the COM-B model (ie, capability, opportunity, and motivation). Detailed results on the barriers and facilitators are presented in Table 2. Briefly, barriers to COVID-19 vaccination could be grouped into those related to capability (eg, vaccine misinformation), opportunity (eg, limited access to the vaccines and poorly planned vaccination drives), and motivation (eg, public concerns related to the vaccines). Similarly, facilitators of COVID-19 vaccination could be grouped into those related to capability (eg, access to accurate information related to the vaccines and having the right approach in delivering public education), opportunity (eg, sufficient access to the vaccines and well-planned vaccination drives), and motivation (eg, public perception about the threat of COVID-19 and public optimism about the vaccines).

**Figure 3:**
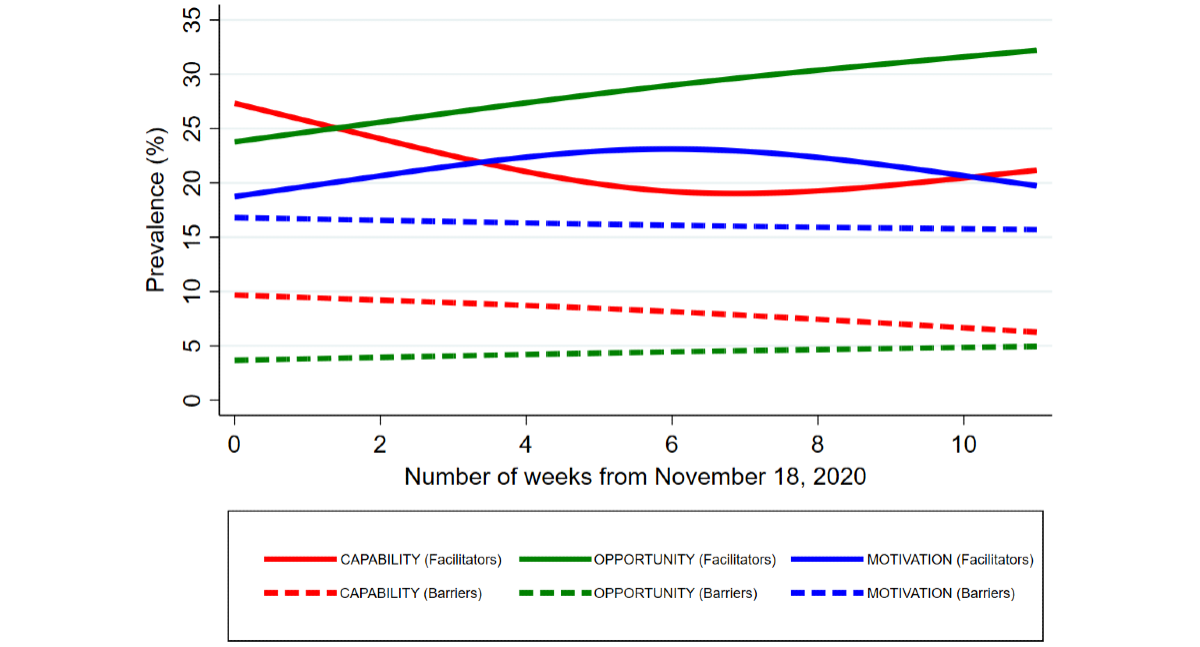
Temporal variations in the prevalence of the barriers and facilitators related to each component in the COM-B model. The graph was plotted using restricted cubic spline smoothing.

Figure 3 shows the variations in the prevalence of barriers and facilitators related to each component in the COM-B model (ie, capability, opportunity, and motivation) over the 11-week study period. In general, tweets related to facilitators had a higher prevalence than those related to barriers. Facilitators related to capability were highly talked about in the initial weeks after the press release regarding the Pfizer-BioNTech vaccine, followed by a declining trend in the subsequent weeks. Facilitators related to opportunity rose drastically over the 11 weeks, while facilitators related to motivation peaked around the sixth week. In contrast, tweets related to barriers remained largely constant throughout the study period, with those related to motivation being more prevalent than those related to capability or opportunity.

**Table 2:** Barriers and facilitators of COVID-19 vaccination, grouped according to the three components of the COM-B model.

COM-B model^a^ component	Barriers	Facilitators
Capability^b^	Inaccurate information related to COVID-19 vaccines: Vaccine misinformation (Topic 26) Politicians’ claims about COVID-19 as a hoax (Topics 17 and 18) The antivaccine movement and conspiracy claims (Topic 55)	Access to accurate information related to COVID-19 vaccines: Accurate news reporting related vaccine development and approval (Topics 1, 10, 13, 23, and 25) Accurate news reporting related to effectiveness of the vaccines against new strains of the COVID-19 virus (Topic 52) Accurate news reporting on the impact of COVID-19 (Topics 35 and 56) Having the right approach to delivering public education on COVID-19 vaccines: Involving an expert panel in public education on COVID-19 vaccines (Topic 8) Using various media (eg, radio and video) in public education on COVID-19 vaccines (Topics 9 and 22) Providing simple explanations about vaccine-related issues (eg, efficacy of the vaccines, the mechanisms of how the vaccines may work, and how the vaccines can help in achieving herd immunity; Topics 7, 20, 34, and 51)
Opportunity^c^	Limited access to COVID-19 vaccines: Insufficient supply of COVID-19 vaccines (Topic 12) Inequitable access to the vaccines (Topic 33) Poorly planned vaccination drives: Delays in vaccination rollout (Topic 31)	Having sufficient access to COVID-19 vaccines: Ensuring equitable access to the vaccines (eg, ethical principles of vaccine allocation and equitable access across various populations; Topics 3, 15, 21, and 39) Ensuring timely shipment of the vaccines (Topic 49) Having a coordinated distribution plan for the vaccines (Topics 38 and 57) Having a well-planned vaccination drive: Defining priority groups for vaccination, (eg, frontline workers, long-term care homes, and individuals with high-risk health conditions; Topics 4, 28, 45, and 46) Engaging employers in facilitating COVID-19 vaccination (Topic 24) Using appointment systems and multiple vaccination sites in vaccination rollouts (Topics 29 and 48) Having a coordinated national plan for vaccination drives (Topics 32 and 44)
Motivation^d^	Public concerns related to COVID-19 vaccines: Public concerns about death related to COVID-19 vaccines (Topic 19) Public concerns about the impact of COVID-19 vaccines on fertility (Topic 11) Concerns about COVID-19 vaccine safety in pregnancy and the vaccines’ use in children (Topic 43) Public concerns about adverse events related to COVID-19 vaccines (Topics 5 and 50) Public concerns about fitness for vaccination among individuals with medical conditions or allergies (Topic 6) Public concerns about short development phases of COVID-19 vaccines (Topic 16) Public concerns about the effectiveness of COVID-19 vaccines against new strains of COVID-19 (Topic 53) Public concerns about vaccine scams regarding mining of personal and financial information (Topic 54) Public concerns about COVID-19 causing false-positive results on HIV tests (Topic 41) Mistrust among minority communities with regard to COVID-19 vaccination (Topic 36)	Public perception about the threat of COVID-19: Voicing the importance of staying vigilant to keep control of COVID-19 (Topic 59) Comparing COVID-19 and COVID-19 vaccination statistics to drive vaccination efforts (Topics 2, 27, and 47) Public optimism about COVID-19 vaccines: Hoping for COVID-19 vaccination in making the days ahead better (Topic 37) Sharing of positive emotions related to receiving COVID-19 vaccines (Topic 30) Feeling positivity regarding contributing to COVID-19 vaccination development (Topics 40 and 42) Influence of public figures in instilling public trust (Topics 14 and 58)

^a^The COM-B (capability, opportunity, and motivation components of behavior) model provides a framework to understand and change human behaviors in the context of public health.

^b^Capability refers to an individual’s psychological and physical capacity to make a behavior possible, such as having the necessary knowledge and skills to perform a target behavior.

^c^Opportunity refers to attributes that lie outside an individual, physically and socially, that make a behavior possible, such as environmental factors or social and cultural norms.

^d^Motivation refers to the automatic or reflective mental processes that energize and direct behavior, which can include a conscious thought process in deciding on a behavior or a less conscious thought process driven by desires or habits.

## Discussion

### Principal Findings

This study used social media data to capture real-time public conversations about COVID-19 vaccines after November 18, 2020, following a press release regarding the first effective vaccine. Six main themes could be identified from the tweets: (1) emotional reactions related to COVID-19 vaccines, (2) public concerns related to COVID-19 vaccines, (3) discussions about news related to COVID-19 vaccines, (4) public health communications about COVID-19 vaccines, (5) discussions about the approach to COVID-19 vaccination drives, and (6) discussions about the distribution of COVID-19 vaccines. Tweets with negative sentiments largely fell within the themes of emotional reactions and public concerns related to COVID-19 vaccines. The tweets could be further classified using the COM-B model to examine the barriers and facilitators related to COVID-19 vaccines. Tweets that focused on barriers remained largely constant throughout the study period, with those related to motivation (eg, public concern) being more prevalent than those related to capability (eg, misinformation) or opportunity (eg, vaccine inaccessibility and poorly planned vaccination drive). In contrast, tweets that focused on facilitators showed temporal variations over the 11-week study period: those related to capability (eg, access to accurate information and public education) peaked initially, while those related to motivation (eg, perceived threats from COVID-19 and optimistic attitudes) peaked around the sixth week and those related to opportunity (eg, sufficient supply and well-planned vaccination drives) rose drastically over time.

### Using Social Media Data to Inform Vaccination Strategies

**Figure 4:**
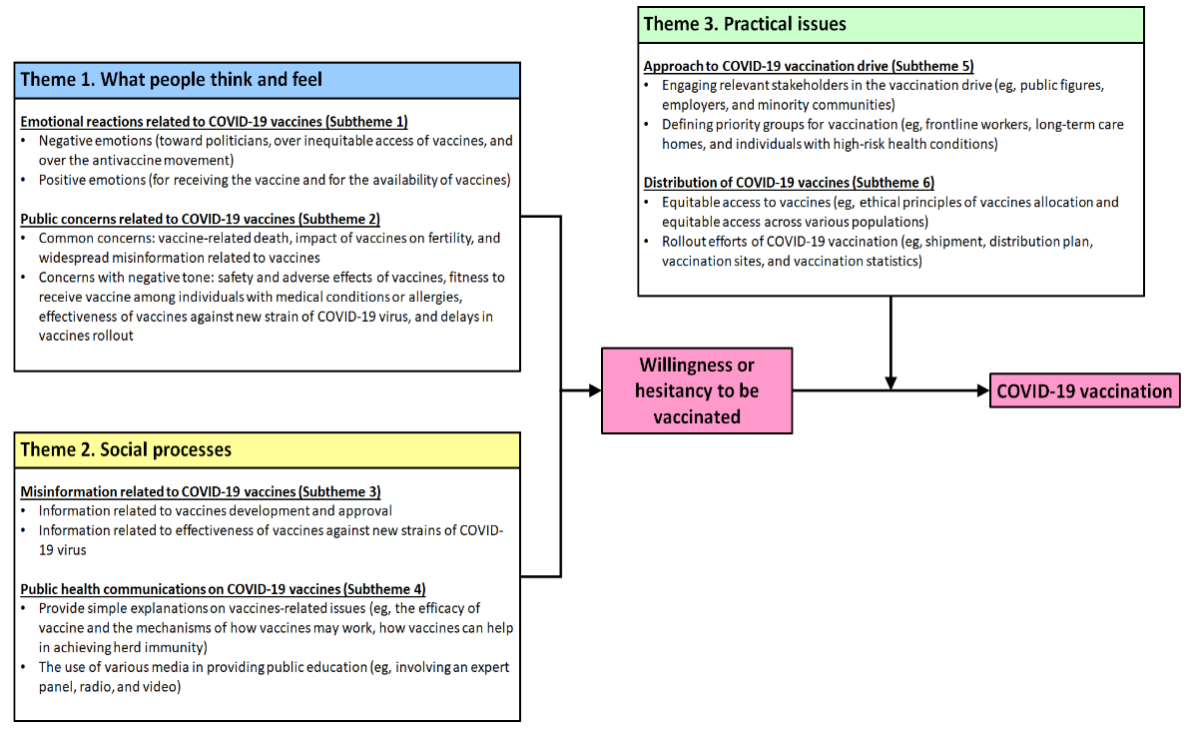
An explanatory model regarding the key drivers of COVID-19 vaccination, expanding on the original Increasing Vaccination Model.

Our findings on the six themes (Table 1) are consistent with recent literature related to individuals’ motivations to receive vaccines and may potentially have policy implications for improving COVID-19 vaccine uptake. In 2018, the WHO convened an expert working group [8] to track and address the challenge of undervaccination that is often prevalent around the world. By adapting from prior literature [7], the working group published a theoretical Increasing Vaccination Model (IVM) [8] to clarify key processes that may affect whether an individual receives a vaccine. At the heart of this model, the WHO highlighted that individuals’ motivations to be vaccinated are shaped by what they think and feel (eg, perceived risks and benefits of vaccination), as well as by social processes that play out in their environment (eg, strong recommendation from health care providers and misinformation that circulates within their social networks) [8]. Eventually, practical issues (eg, vaccine availability, accessibility, cost, and convenience) will further shift the individuals from being willing to be vaccinated to actually receiving the vaccines. It is notable that the six themes from this study possibly align well with the WHO’s IVM and may potentially expand on the key processes that are specific to the context of COVID-19 vaccination. For instance, our first two themes provide real-time examples regarding what people think and feel toward COVID-19 vaccination, while the third and fourth themes highlight the social processes that are currently in play in the community (eg, circulating news items that have captured public attention and ongoing public health communications about COVID-19 vaccines). Similarly, the fifth and sixth themes possibly exemplify vaccine-related practical issues that have captured public attention, including those related to vaccine distribution and vaccination drives. Such mapping, between our six themes and the three key processes from the IVM, is further illustrated in Figure 4. Given the expected rollout of COVID-19 vaccination globally, this expanded model in Figure 4 may possibly provide richer contextual details on the key processes that are relevant to successful COVID-19 vaccination drives. For example, in the planning of COVID-19 vaccination, Figure 4 highlights the need for policy makers to be mindful of common emotions and concerns in the community, which may then be addressed through targeted public health communications as well as through cautious debunking of misinformation. In addition, Figure 4 also elaborates on practical issues that need to be addressed in vaccination drives, such as engagement of stakeholders, clarification of priority groups for vaccination, and equitable access to the vaccines.

Our findings on the barriers and facilitators to COVID-19 vaccination (Table 2 and Figure 3) present a different, yet equally relevant, set of information to policy makers. Figure 3 provides feedback to policy makers on the evolving barriers and facilitators in ongoing COVID-19 vaccination and, hence, may allow policy makers to further tweak their policies given these close-to-real-time ground sentiments. For example, the findings from Figure 3 may possibly assure policy makers that the facilitators of COVID-19 vaccination have largely been put in place, with these facilitators outweighing the various barriers to vaccination in the community. Yet, at the same time, Figure 3 also highlights a disquieting trend: barriers related to individuals’ motivations to receive COVID-19 vaccines have remained prominent and unchanging across time and may be an area that requires further interventions by policy makers. Given that the barriers related to motivation were largely driven by public concerns regarding COVID-19 vaccines, such as those related to vaccine safety as seen in Table 2, policy makers may potentially adopt some of the proposed interventions related to the COM-B model [28] to address these prevailing public concerns, which have been highlighted in Table 2. As an illustration, barriers related to motivation in the COM-B model can often be modified through persuasion (ie, using communication to stimulate action), modeling (ie, providing an example for people to aspire to or imitate), and incentivization (ie, creating an expectation of reward) [28]. Policy makers may possibly intensify the use of these three approaches in modifying barriers related to motivation, such as by convincing the public of the safety and effectiveness of COVID-19 vaccines (ie, persuasion) [46], broadcasting special events with public figures or celebrities receiving the vaccines (ie, modeling) [47,48], and lifting COVID-19 restrictions related to social gatherings among those who have received the vaccines (ie, incentivization) [49].

### Broader Roles of Social Media in COVID-19 Vaccination

**Figure 5:**
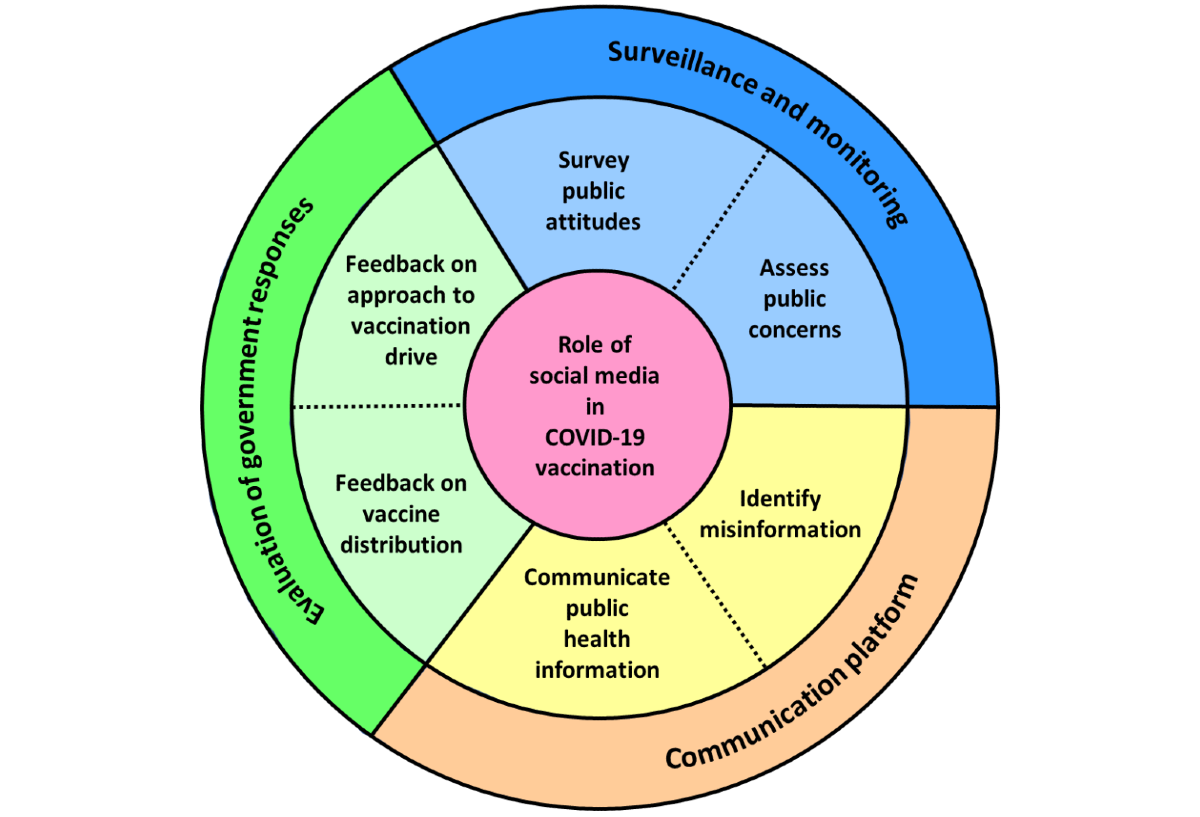
A framework for the role of social media in COVID-19 vaccination.

From a broader perspective, findings from this study demonstrated the potential roles of social media in ongoing COVID-19 vaccination drives. Social media has been increasingly recognized in recent literature as a useful source of data to inform issues of public health interest [50] given the nature of social media data, which reflect real-time ground sentiments, as well as the potential utility of social media platforms for mass dissemination of health-related information [9]. The relevance of social media data became more apparent during the COVID-19 pandemic, whereby many pandemic-related issues were fluid in nature, such as those related to infection rate, government policy, and vaccination plans; hence, there is a constant need to disseminate accurate information related to the pandemic in a timely manner [9]. The potential roles of social media during the COVID-19 pandemic were further highlighted in a recent scoping review [50], whereby six generic roles of social media could be identified, as follows: surveying public attitudes, assessing mental health, detecting COVID-19 cases, identifying misinformation, evaluating the quality of public health communications, and analyzing government responses to the pandemic [50]. Although not all of the generic roles are applicable to the specific context of COVID-19 vaccination, some of these roles are exemplified by findings from this study. For example, the role of social media in surveying public attitudes is epitomized by our first two themes, which further clarified the emotional reactions and public concerns related to COVID-19 vaccines. Meanwhile, the role of social media in identifying misinformation may possibly be seen in our third theme. News items related to COVID-19 vaccines are constantly discussed on social media, and during this process, inaccurate or false information may potentially be communicated. Consequently, social media may be used to identify real-time misinformation that has been widely circulated in public. Similarly, the role of social media in evaluating the quality of public health communications is illustrated by our fourth theme, whereby social media posts may be surveyed to examine the prevailing approach and content in public health communications related to COVID-19 vaccines. Likewise, the role of social media in analyzing government responses to the pandemic is seen in our fifth and sixth themes, whereby social media posts may be used to gain real-time public feedback on operational issues related to vaccination drives. By consolidating prior evidence [50] and findings from this study, the role of social media—specific to COVID-19 vaccination—may possibly be summarized in the framework in Figure 5. In essence, we propose that social media may play at least three key roles in ongoing COVID-19 vaccination drives, as follows: surveillance and monitoring of public concerns regarding COVID-19 vaccines, a platform for accurate communication of vaccine-related information, and evaluation of government responses in the vaccine rollout.

### Limitations

Some limitations should be considered in this study. First, Twitter posts were used to exemplify social media data. Although Twitter is among the most widely used social media platforms [9,21], it may not fully represent users of other social media platforms. Second, we only included tweets that were posted in English due to challenges in analyzing posts in different languages together. Hence, our findings may be more representative of English-speaking populations.

Third, most of Twitter’s users were from North America and Europe. Keeping this in mind, our findings may not be as generalizable to other countries. Fourth, the findings may not fully represent the perspectives of the wider population, given that social media is largely used by individuals who have internet access and are technology savvy [51].

Fifth, previous research has found that nonhuman Twitter users (ie, bots) may artificially manipulate public opinion on social media [52]. Most of such nonhuman tweets would have been excluded in this study, as we selected only tweets by users with actual human names and we excluded retweets and duplicate tweets. Notwithstanding these efforts, a small number of these nonhuman tweets could possibly still have remained in the study sample.

Sixth, insofar as unsupervised machine learning is well suited to analyses of large volumes of free-text data [24], such analyses may not be as in depth as manually conducted qualitative analyses. To address this limitation, the output from machine learning was further refined by manual analyses by the two authors using currently recommended best practices in qualitative studies [53]. 

Seventh, the sentiment analysis in this study was conducted to supplement the main findings from topic modeling. Although the results from our sentiment analysis have face validity (ie, consistently highlighting topics with negative sentiments, as seen in Table 1) and our approach to sentiment analysis, using the VADER package, has good supporting evidence from the literature [36,42,43], readers should be mindful that sentiment analysis remains an evolving area in the field of natural language processing and, hence, the findings from sentiment analysis should probably be treated as exploratory in nature. It is also possible that other newer techniques of sentiment analysis, especially those based on supervised deep learning, may achieve better accuracy in sentiment analysis. However, the development of new models for sentiment analysis is probably a separate area that may benefit from further research and may be beyond the scope of this study.

### Conclusions

In conclusion, this study used unsupervised machine learning to identify six overarching themes related to COVID-19 vaccines on social media, of which some themes contained tweets with more negative sentiments. The findings may facilitate the formulation of comprehensive strategies to improve COVID-19 vaccine uptake in the community; they highlight the key processes that require attention by policy makers in the planning of COVID-19 vaccination and provide feedback on the evolving barriers and facilitators in ongoing vaccination drives to allow further policy tweaks. From a broader perspective, the findings may also be consolidated into a framework to illustrate three key roles of social media in COVID-19 vaccination, as follows: surveillance and monitoring, a communication platform, and evaluation of government responses.

## Appendix

Multimedia Appendix 1 Word clouds for the six themes related to COVID-19 vaccination. The word clouds have been weighted using term frequency–inverse document frequency to give more emphasis to words that are unique to each tweet.

## Abbreviations

COM-B: capability, opportunity, and motivation components of behavior
IVM: Increasing Vaccination Model
VADER: Valence Aware Dictionary and Sentiment Reasoner
WHO: World Health Organization
