# Supplementary material for: Examining the Utility of Social Media in COVID-19 Vaccination: Unsupervised Learning of 672,133 Twitter Posts
Source: JMIR Public Health Surveill. 2021 Nov 3;7(11):e29789. doi: 10.2196/29789 (PMC8568045; doi:10.2196/29789)
Supplement: Multimedia Appendix 1 [file publichealth_v7i11e29789_app1.docx]

**SUPPLEMENTARY MATERIAL**

**Multimedia Appendix 1**. Word clouds for the six themes related to COVID-19 vaccination. The word clouds have been weighted using Term Frequency–Inverse Document Frequency (TF-IDF) to give more emphasis to words that are unique to each tweet.

| **Theme 1**. Emotional reactions related to COVID-19 vaccine  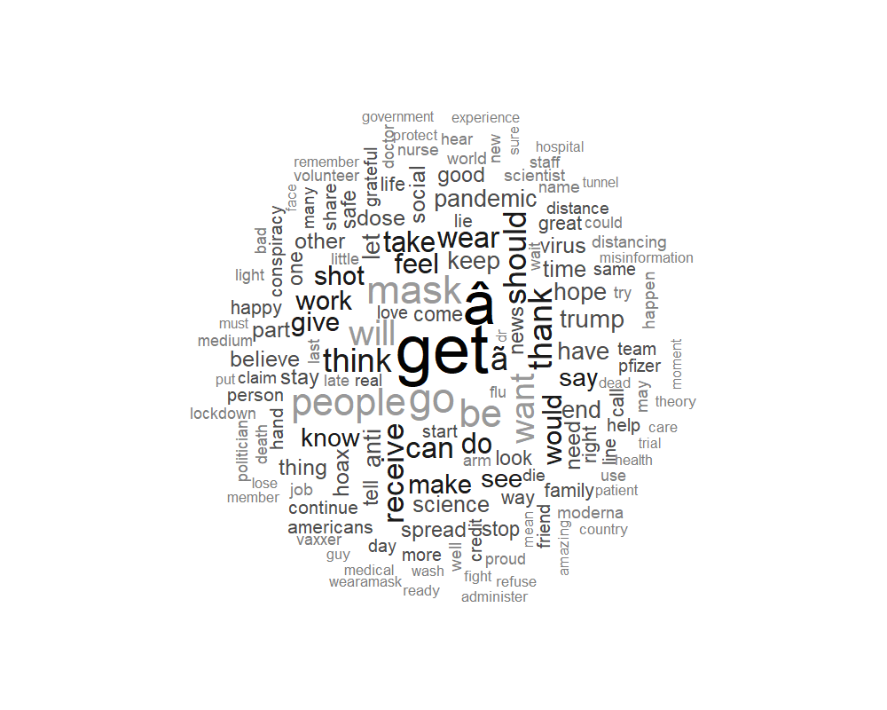 | **Theme 2**. Public concerns related to COVID-19 vaccine  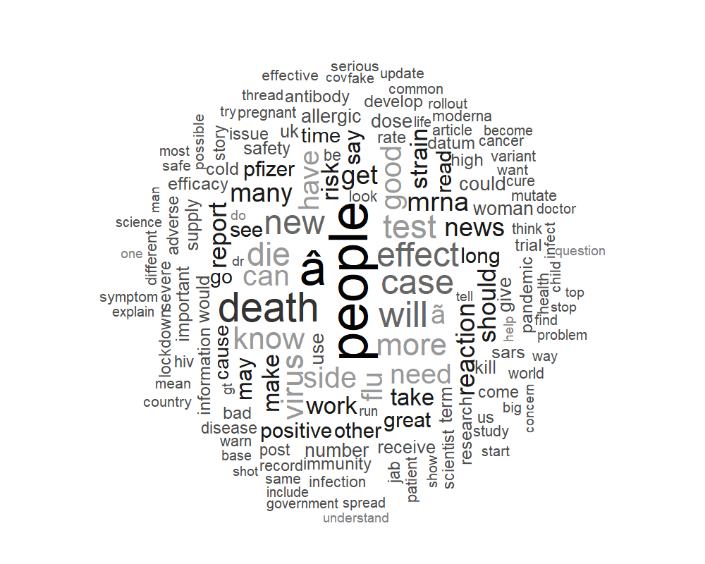 |
| --- | --- |
| **Theme 3**. Discussions on news related to COVID-19 vaccine  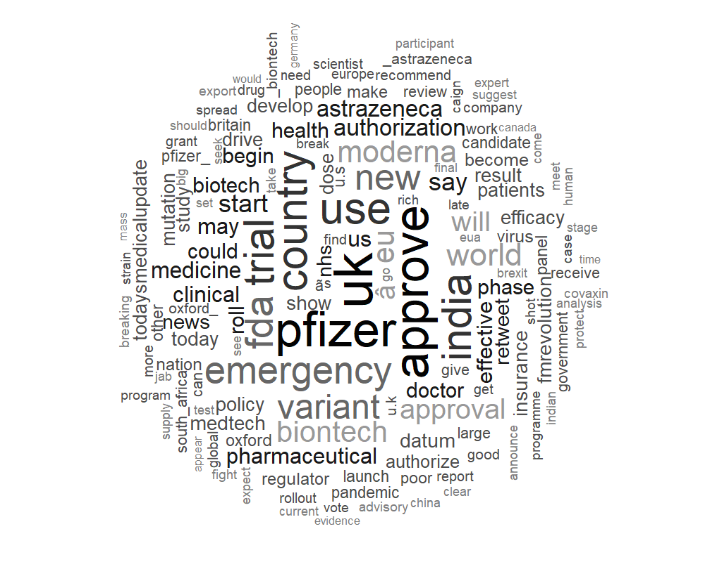 | **Theme 4**. Public health communications on COVID-19 vaccine  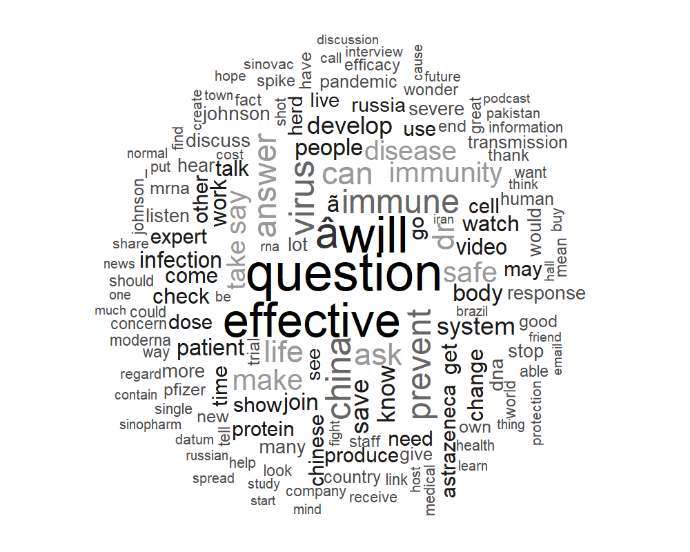 |
| **Theme 5**. Discussions on the approach to COVID-19 vaccination drive  **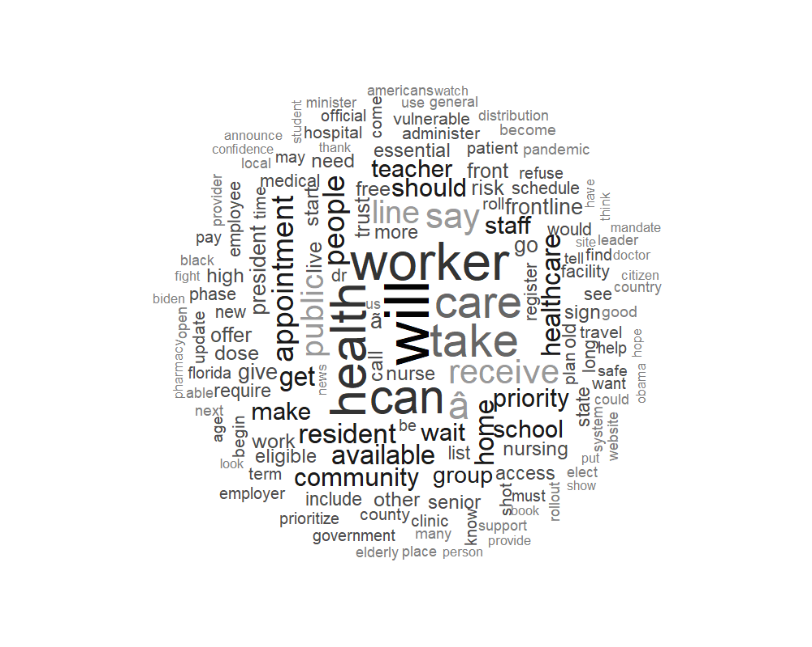** | **Theme 6**. Discussions on the distribution of COVID-19 vaccine  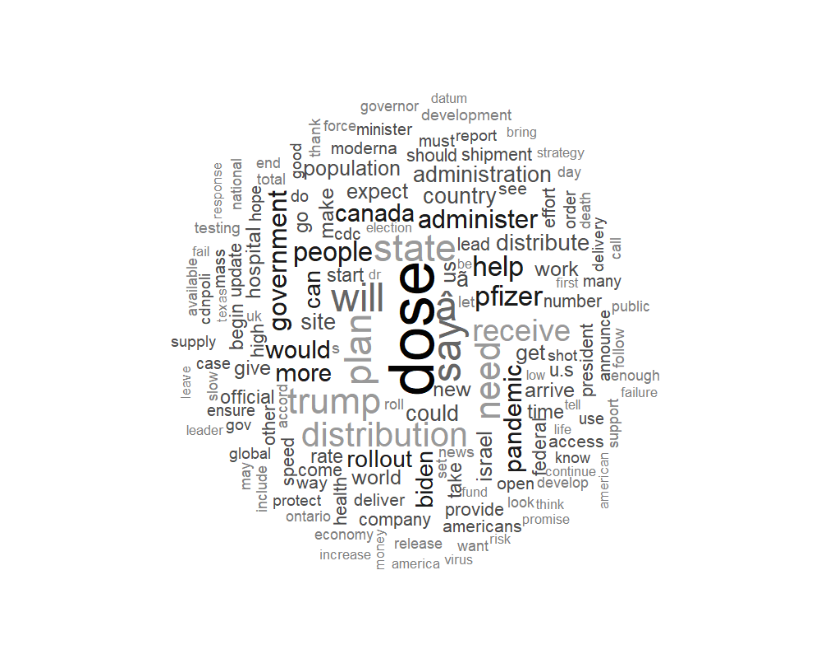 |
